# Cancer stem cells in basic science and in translational oncology: can we translate into clinical application?

**DOI:** 10.1186/s13045-015-0113-9

**Published:** 2015-02-25

**Authors:** Axel Schulenburg, Katharina Blatt, Sabine Cerny-Reiterer, Irina Sadovnik, Harald Herrmann, Brigitte Marian, Thomas W Grunt, Christoph C Zielinski, Peter Valent

**Affiliations:** Bone Marrow Transplantation Unit, Department of Internal Medicine I, Medical University of Vienna, Währinger Gürtel 18-20, Vienna, A-1090 Wien Austria; Ludwig Boltzmann Cluster Oncology, Medical University of Vienna, Spitalgasse 23, Vienna, 1090 Wien Austria; Department of Medicine I, Division of Hematology and Hemostaseology, Medical University of Vienna, Währinger Gürtel 18-20, Vienna, 1090 Wien Austria; Department of Radiation Therapy, Medical University of Vienna, Spitalgasse 23, Vienna, 1090 Wien Austria; Department of Medicine I, Institute for Cancer Research, Medical University of Vienna, Währinger Gürtel 18-20, Vienna, 1090 Wien Austria; Department of Medicine I, Division of Clinical Oncology, Medical University of Vienna, Währinger Gürtel 18-20, Vienna, 1090 Wien Austria; Department of Medicine I, Stem Cell Transplantation Unit, Medical University of Vienna, Waehringer Guertel 18-20, A-1090 Wien, Austria

**Keywords:** Cancer stem cells, Targeted therapy, Drug resistance

## Abstract

Since their description and identification in leukemias and solid tumors, cancer stem cells (CSC) have been the subject of intensive research in translational oncology. Indeed, recent advances have led to the identification of CSC markers, CSC targets, and the preclinical and clinical evaluation of the CSC-eradicating (curative) potential of various drugs. However, although diverse CSC markers and targets have been identified, several questions remain, such as the origin and evolution of CSC, mechanisms underlying resistance of CSC against various targeted drugs, and the biochemical basis and function of stroma cell-CSC interactions in the so-called ‘stem cell niche.’ Additional aspects that have to be taken into account when considering CSC elimination as primary treatment-goal are the genomic plasticity and extensive subclone formation of CSC. Notably, various cell fractions with different combinations of molecular aberrations and varying proliferative potential may display CSC function in a given neoplasm, and the related molecular complexity of the genome in CSC subsets is considered to contribute essentially to disease evolution and acquired drug resistance. In the current article, we discuss new developments in the field of CSC research and whether these new concepts can be exploited in clinical practice in the future.

## Introduction

The principle concept of cancer stem cells (CSC) has gained increasing acceptance in recent years [1-10]. By definition, CSC exhibit self-renewal activity and long-term cancer-propagating capacity [1-9]. By contrast, more mature clonal cells in the same neoplasm have limited proliferative potential. In leukemias, CSC are also known as leukemic stem cells (LSC) [5,8,11-18]. The concept of neoplastic stem cells may provide explanations for the failure of various cytoreductive agents to produce long-lasting responses in patients [1-9,11,15-18]. Notably, in many instances, anti-neoplastic drugs act on more mature neoplastic cells rather than CSC/LSC, a phenomenon that is explained in part by the fact that these cells exhibit intrinsic resistance [19-23]. Moreover, CSC often develop acquired drug resistance and thus produce more malignant subclones over time [11,24-26].

All these observations point to the need to develop new CSC-eliminating treatment strategies through which cure rates and survival can be improved [16,27-31]. In other words, CSC have been recognized as a major ‘target cell population’ in oncology in recent years, and considerable effort has been made to identify novel CSC markers and target expression profiles and to measure responses of these cells to various targeted drugs.

The present article provides a summary of our knowledge on CSC/LSC, with special focus on the possibility to translate CSC/LSC-targeting treatment concepts into clinical application. Unless otherwise stated, this article refers to CSC/LSC in primary human malignancies. With regard to cell line models and engineered CSC-like cells or other more ‘artificial’ models that may also support CSC research, we refer to the published literature.

### Definition and function of cancer stem cells

In contrast to more mature cancer cells, CSC are self-renewing cells with long-term proliferative potential [1-9,11]. As a result, CSC can maintain a given neoplasm for prolonged time periods. In most cases, these cells can also produce a cancer (CSC) or leukemia (LSC) in immunodeficient mice (xenotransplantation model) which enables their detection and quantification [1-9,32,33]. Previous studies have used non-obese diabetic mice with severe combined immunodeficiency (NOD/SCID) [1-3,5,6,32,33]. In several tumor models, this mouse strain is a sufficient or even a preferable model to study CSC biology [34]. However, more recent data suggest that in several primary malignancies, NOD/SCID with loss-of-function-mutated IL-2Rgamma chain or IL-2Rgamma chain-knock out NOD/shi-SCID mice (NSG or NOG mice) provide superior engraftment rates [35-38]. Therefore, many current studies on primary CSC/LSC employ NSG mice. Depending on the type of disease, neoplastic cells are injected intravenously, subcutaneously, or directly into solid organs (orthotopic application) [27,39-44]. An important point is that ‘short-term engraftment’ (or just simple maintenance) of tumor/leukemic cells has to be differentiated from long-term engraftment, only the latter being indicative of the presence of functionally active (self-renewing) CSC. Long-term engraftment and growth of cancers/leukemias is best demonstrable by recovering engrafted cells from primary recipient mice and injecting these cells into secondary recipient animals [32,39,40,42,43,45-47].

Despite advanced technologies and novel mouse models, xenotransplantation assays for human CSC have several limitations. First, the microenvironment is often species-specific or tumor-specific. Second, in a neoplasm with low growth-rate (for example, indolent/low-grade/chronic tumor; premalignant neoplasms), the development phase of the neoplasm may exceed the lifetime of a mouse. Moreover, the CSC pool is composed of heterogeneous populations of tumor-initiating cells with subclone-specific molecular properties and varying growth characteristics *in vivo* [11,25,26,28,48]. Some of the CSC may be recognized (and eliminated) by the residual immune system of xeno-transplanted mice [37,38]. On the other hand, the lack of a natural immune system and thus tumor immune surveillance in highly immunodeficient mice may facilitate the uncontrolled expansion of clinically irrelevant sub-clones. Therefore, several attempts are currently made to establish NSG-mouse models harboring a human immune system.

A frequently discussed alternative to *in vivo* xenotransplantation studies are *in vitro* long-term culture experiments to study the growth and maintenance of CSC [47,49-53]. Although helpful as a screen approach, these assays are not sufficient for evaluating the *in vivo* self-renewal capacity of ‘true’ CSC. Several *in vitro* assays employ stromal cells which may provide some of the ‘niche-factors’ required for long-term growth CSC [47,49-53]. Solid tumor cells often grow in ‘spheres’ or clusters for prolonged time periods in such assays [47,49-53]. However, as mentioned above, the available *in vitro* assays cannot replace *in vivo* xenotransplantation models when long-term self renewal and tumor propagation should be examined.

### Identification and enrichment of CSC/LSC

Several different approaches, through which CSC/LSC can be identified and enriched in primary cancer/leukemia samples, have been developed in the past [1-3,5-7,9,11-13,27,54-61]. A widely applied strategy is to use antibodies directed against certain cell surface antigens that are (or are not) expressed on CSC [1-3,5-7,9,11-13,27]. Expression of surface antigens is best determined by multicolor flow cytometry. Enrichment of CSC/LSC can be performed by fluorescence-activated cell sorting (FACS) or magnetic cell sorting [1-9,13,15-18,62-69]. Both techniques have certain limitations. One general problem is that the ‘so-called’ stem cell markers are often not specific for CSC or LSC. Likewise, the stem cell-related antigen CD34 is not only expressed on hematopoietic stem cells but also on myeloid progenitor cells and endothelial cells, and KIT is not only expressed on hematopoietic stem- and progenitor cells but also on mast cells, germ cells, and melanocytes [70,71]. Therefore, it is essential to apply combinations of antibodies when detecting and analyzing CSC/LSC in various tissues. Usually, one or two organ-specific markers are employed to confirm the primary origin of cells (Tables 1 and 2). The pan-hematopoietic marker CD45 is widely used to confirm the hematopoietic origin of cells or to exclude leukocytes in primary fractions obtained from solid tumors. Additional antibodies are applied to delineate CSC from more mature neoplastic cells [1-3,5-7,9,11-13,27,65-69,72,73]. In case of myeloid leukemias, the antigen profiles of more mature cells are well defined, and the approach to deplete these (Lin+) cells from LSC is well established. However, in certain leukemias, LSC may aberrantly express one or even several of the ‘lineage-related’ antigens. In such leukemias, application of the ‘Lin-cocktail’ may lead to a loss of LSC subsets. Another problem is that antibody-bound cells may be detected and eliminated by the residual immune system of NOD/SCID mice. This problem has been outlined in acute myeloid leukemia (AML) where CD38+ cells (CD38 antibody-laden) may be cleared by the residual immune system of NOD/SCID mice [38]. The problem has been addressed by switching from NOD/SCID mice to NSG (or NOG) mice that lack a functionally active cytokine receptor gamma chain [35-38]. As mentioned above, the lack of a natural immune system in these models is a remaining issue that will hopefully be solved by introducing a humanized immune system into these mice. Another caveat is that some of the antibody preparations used to define CSC may induce apoptosis in cancer cells [74].

**Table 1:** **Phenotype of neoplastic stem cells (NSC) in hematologic neoplasms**

**Neoplasm**	**Defined cell subsets containing NSC**	**Cell surface antigens aberrantly expressed or overexpressed on neoplastic SC**
AML	CD34+/CD38− [32]	CD25 [75], CD33 [76], CD52[77]
CD96 [68], CD123 [69]		
		CLL-1 [67]
AML	CD34+/CD38+ [38]	n.k.
AML_NPM1mutated_	CD34− blast-like [78]	n.k.
MDS	CD34+ [79]	CD123 [80]
MDS with 5q-	CD34+/CD38− [81]	CD52 [77], CD123
MPN	CD34+ [82]	n.k.
CML CD123 [86], IL-1RAP [87]	CD34+/CD38− [17]	CD25 [83], CD26 [84], CD33 [85]
Ph + ALL	CD34+/CD38−/CD19+ [88]	CD25, CD26^a^, CD52
Ph − ALL	CD34+/CD19+ [89]	n.k.
CLL	CD34+/CD19+ [90]	CD5
Myeloma	CD20+/CD27+/CD138− [91]	n.k.

AML, acute myeloid leukemia; MDS, myelodysplastic syndrome(s); MPN, myeloproliferative neoplasm(s); CML, chronic myeloid leukemia; ALL, acute lymphoblastic leukemia; CLL; chronic lymphocytic leukemia; n.k., not known; NSC, neoplastic stem cells; SC, stem cell; Ph+, Philadelphia chromosome-positive; Ph−, Philadelphia chromosome-negative; IL-1RAP, interleukin-1 receptor accessory protein. ^a^In a subset of patients with Ph + ALL, LSC express CD26.

**Table 2:** **Phenotype of CSC-enriched fractions of neoplastic cells in solid tumors** ^a^

**Neoplasm**	**Phenotype of CSC-rich cell fraction**	**Reference**
Breast cancer	CD326+/CD45−/CD44+/CD24−	[40]
	CD44+/CD49f+/CD133+	[92]
	CD326+/CD44+/CD47+/MET+	[93]
	CD29f	
Gastric cancer	CD326+/CD44+	[94]
	CD49f+	[95]
	CD90+	[96]
	LGR5^b^+	[97]
	CD44+	[98]
Colon cancer	CD326+/CD44+/CD166+	[99]
	CD44+/CD49f+/CD133+	[100]
	LGR5^b^+	[101]
	CD133+	[47]
SCLC	CD133+	[102]
NSCLC	CD133+	[103]
Pancreatic cancer	CD44+/CD24+/CD326+	[43]
	CD133+/CXCR4+	[39]
HCC	CD326	[104]
	CD133+	[105]
	CD44+/CD90+	[44]
Glioblastoma	CD133+	[42]
	CD15+/CD133+	[106]
	CD15+/CD133+	[106]
	CD133+/SSEA-1+	[107]
Ewing’s sarcoma	CD133+	[108]
Osteosarcoma	CD133+	[109]
	CD117+/STRO-1+	[110]
	CD271+	[111]
Ovarian cancer	CD24+/CD44+/CD326+	[112]
	CD44+/CD117+	[113]
	CD133+	[114]
Prostate cancer	CD44+/CD49f/CD326+	[115]
	CD44+/CD24−	[116]
	CD44+/CD133+	[117]
Melanoma	CD271+	[118]
	ABCB5+	[119]
	EPOR+	[120]

NSC, neoplastic stem cells; LGR5, Leucine-rich repeat-containing G-protein coupled receptor 5; SCLC, small cell lung cancer; n.k., not known; NSCL, non-small cell lung cancer; HCC, hepatocellular carcionoma; EPOR, erythropoietin receptor. ^a^Expression of NSC markers refers to primary human cells tested in xenotransplantation assays and/or in a sphere-formation assay. ^b^LGR5 is not detectable on human NSC by flow cytometry.

In solid tumors, a general problem is that for most neoplasms, robust markers discriminating between more mature and immature cells are not available. In colorectal cancer and some other solid tumors, the Wnt target gene LGR5 has been described as a potential CSC marker [121,122]. Other markers, such as CD44, are broadly expressed on tumor cells and also in other cell types (for example, leukocytes) present in the same organ sites. Another problem is that several CSC-homing receptors and their ligands are species specific which may prevent homing of CSC to their specific microenvironment (CSC niche) in mice. Such limitations can be overcame by direct (orthotopic) injection of CSC into target organs or into tissue scaffolds [39-44,46,123,124]. Other potential solutions may be to co-transplant ‘niche-relevant’ autologous (human) stroma cells together with CSC, to treat mice with cytokines promoting the growth of CSC/LSC or to employ NSG mice engineered to express human niche-associated cytokines such as stem cell factor (SCF) [125]. For the future, mouse models harboring a human immune system as well as human stromal cells might be desirable for studying CSC biology.

Probably the most important problem regarding CSC-recognition is stem cell plasticity and disease heterogeneity [9,11,25,28,54,126]. Likewise, depending on the subtype of myeloid leukemia, LSC may reside within the CD34+/CD38− fraction of the clone but also in the CD34+/CD38+ or even in CD34− cell populations [38,78,126]. It has also been described that LSC may be composed of CD133+ and CD133− subfractions [64,127]. Only a few markers, such as CLL-1 or interleukin-1 receptor accessory protein (IL-1RAP), may be more or less specific for LSC in certain human leukemia models [67,87]. These markers are interesting tools and may serve as diagnostic markers or/and therapeutic targets in the future. Tables 1 and 2 show a summary of markers expressed on CSC in hematopoietic neoplasms (Table 1) and non-hematologic malignancies (Table 2).

### Regulation of growth and development of CSC/LSC

So far, little is known about the regulation of growth and survival of CSC/LSC in hematopoietic and non-hematopoietic malignancies. The development phase of CSC may often last for years if not decades [28,48,54,128]. In an early phase (pre-phase) of cancer or leukemia development, neoplastic stem cells may be slowly cycling cells that produce small-sized subclones [11,28,54,128]. At this early hypothetical stage of cancer evolution, it may be preferable to call these cells premalignant neoplastic stem cells (NSC) rather than CSC/LSC [24,28,128-131]. Later, when these premalignant cells have accumulated a sufficient number of molecular lesions (defects) and thereby have ‘learned’ how to escape all relevant surveillance mechanisms, their progeny can expand and form an overt malignancy within short time, so that the term malignant NSC (=CSC or LSC in leukemias) is appropriate [28,48,128,130,132] (Table 3). In early phases of NSC evolution (premalignant stage), the mechanisms and molecules regulating growth, survival, and asymmetrical cell division, may be similar if not the same compared to that in normal stem cells. These factors include cytokines and cytokine-receptors, niche-related factors, including stem cell-homing and chemotactic molecules, pro- and anti-apoptotic molecules, and signaling pathways involved in the regulation of self-renewal and proliferation [133-135]. Later, when NSC-derived neoplastic clones expand to an overt malignancy, several ‘physiologic’ mechanisms controlling growth and differentiation of normal (and premalignant neoplastic) stem cells may no longer work to prevent clonal expansion [28,48,128,130,136-138].

**Table 3:** **Classification of neoplastic stem cells (NSC)**

**Defining properties**	**Premalignant NSC**	**Malignant NSC = CSC/LSC**
Self-renewal	Yes	Yes
Cell cycle	Dormant or very slowly cycling	Slowly cycling or more rapidly cycling
Immediate tumor-initiating potential	No^a^	Yes
Long-term tumor-initiating potential	Facultative potential^a^	Yes
Numbers of somatic acquired molecular lesions/mutations	Relatively low	Relatively high
Drug response	Intrinsic resistance (based in part on quiescence)	Intrinsic and often also acquired resistance in malignant subclones

^a^The potential of a NSC to produce a neoplastic condition does not mean that this cell can form a tumor within a certain time period; however, after a certain latency period, when a sufficient number of molecular lesions have been accumulated, these premalignant NSC may transform to fully malignant NSC (=CSC/LSC) that have immediate tumor-initiating capacity *in vivo* in patients as well as in NSG mice. In a subset of patients, premalignant NSC will never convert into fully malignant NSC (= CSC/LSC). NSC, neoplastic stem cells; CSC/LSC, cancer stem cells/leukemic stem cells.

#### Cytokine regulation of NSC (CSC/LSC)

A number of recent data suggest that the cytokine network is involved in the regulation of self-renewal, growth, survival, and differentiation of NSC [64,69,125]. As mentioned above, the cytokines that regulate growth and function of premalignant NSC may be similar or the same as that regulating growth of normal stem cells. Likewise, in myeloid leukemias, NSC/LSC express receptors for various regulators of normal stem cells, including the IL-3 receptor (CD123/CD131), SCF receptor KIT (CD117), or G-CSF receptor (CD114) [64,69,139]. It has also been described that epidermal growth factor (EGF) receptor family members, including HER2, are expressed on epithelial NSC/CSC, such as mammary CSC [140,141]. There is also evidence that insulin-like growth factor (IGF) receptors and fibroblast growth factor (FGF) receptors play an important role in solid tumors and may be expressed on solid tumor CSC [142-144]. At least in leukemias, the cytokine ligands that bind to these receptors trigger proliferation of LSC-enriched cell fractions [139]. Depending on the type and phase of disease, these cytokines also promote differentiation and maturation of LSC. However, most of these cytokines may not cause self-renewal in LSC. Some of these cytokines, such as IL-3, are also produced in clonal cells and may thus act as autocrine growth regulators of LSC [17,87,145,146]. LSC are also considered to respond to various chemokines. In line with this assumption, LSC express chemokine receptors such as CXCR4 [39,147-150]. A clinically important question is whether premalignant NSC or CSC/LSC express receptors for erythropoietin (EPO), granulocyte colony-stimulating factor (G-CSF), or granulocyte/macrophage colony-stimulating factor (GM-CSF). These cytokines are often administered in tumor patients in order to correct disease-related anemia or to accelerate neutrophil production after chemotherapy. In AML as well as in the myelodysplastic syndromes (MDS), NSC/LSC indeed express receptors for G-CSF and sometimes also for GM-CSF [139]. By contrast, NSC/LSC usually do not express EPO receptors in these malignancies. However, the EPO receptor may be expressed on CSC in a few solid tumors as well as in melanoma-initiating cells [120,151-153]. Table 4 shows a summary of cytokine receptors expressed on CSC and LSC in various malignancies.

**Table 4:** **Cytokine/chemokine receptors detectable on neoplastic stem cells (NSC)**

**Malignancy**	**Cytokine receptors expressed on NSC**
AML	IL-2RA [154], IL-3RA [155], G-CSFR [156], FLT3 [157], SCFR/KIT [158], CXCR4 [159]
MDS	G-CSFR [160], SCFR/KIT, CXCR4 [161]
MPN	G-CSFR [160], SCFR/KIT [162], CXCR4 [163]
Ph + CML	IL-2RA [83], IL-3RA [17], G-CSFR [160], GM-CSFR [164], SCFR/KIT [165], IL-1RAP [87], CXCR4 [166]
Ph + ALL	IL-2RA [167], IL-3RA [168], CXCR4 [169]
Myeloma	CXCR4 [170]
Breast cancer	EGFR [171], ERBB2/Her2 [172], FGFR2 [173], TGFßR [174], MET [93]
Gastric cancer	EGFR [175], ERBB2/Her2 [176]
Colon cancer	EGFR [177], CXCR4 [178], IGF1R [179], TGFßR [180]
SCLC	EGFR [181]
Pancreatic cancer	EGFR [182], CXCR4 [39]
HCC	EGFR, IGF1R [183]
Glioblastoma	EGFR [184], PDGFRB [185], CXCR4 [186], TGFßR [187], MET [188]
Ovarian cancer	EGFR [189], ERBB2/Her2 [190], IGF1R, TGFßR [191]
Prostate cancer	CXCR4 [192]
Melanoma	CXCR1 [193], EPOR [120]

AML, acute myeloid leukemia; IL, interleukin; G-CSFR, granulocyte colony-stimulating factor receptor; SCF, stem cell factor receptor; Ph+, Philadelphia chromosome-positive; CML, chronic myeloid leukemia; GM-CSF, granulocyte- macrophage colony-stimulating factor; ALL, acute lymphoblastic leukemia; CLL, chronic lymphocytic leukemia; EGFR, epidermal growth factor receptor; TGFßR, transforming growth factor ß receptor; IGF1R, insulin-like growth factor 1 receptor; SCLC, small cell lung cancer; NSCL, non-small cell lung cancer; n.k., not known; HCC, hepatocellular carcionoma; PDGFR, platelet-derived growth factor receptor; NGFR, nerve growth factor receptor; EPOR, erythropoietin receptor.

#### Oncogenic signaling pathways in NSC (CSC/LSC)

Growth and function of NSC, including self-renewal and malignant expansion, are considered to depend on a complex network of signaling cascades and molecules. Oncogenic signaling is considered to derive from three distinct classes of molecules, i) the driver lesions (primary oncogenic kinases) that are often disease-specific or at least disease-related, like BCR/ABL in chronic myeloid leukemia (CML), ii) broadly expressed mutated oncogenic kinases, and iii) cytokine-activated stem cell kinases that play a role in survival or/and growth of NSC (example: wt KIT in leukemias). The downstream signaling networks of ‘i,’ ‘ii,’ and ‘iii’ are in part overlapping, may often complement each other, and may sometimes even produce synergistic effects on downstream activation and thus oncogenesis [165,194-196]. In an early phase of cancer evolution, the driver mutation (‘i’) and otherwise physiologic mechanisms (‘iii’) may play a predominant role. However, with disease progression, more and more additional oncogenic signaling molecules (‘ii’) and pathways become activated [197-200]. Thus, in advanced phases of a malignancy, additional signaling cascades and networks may play a more and more decisive role in CSC/LSC expansion and resistance. All three classes of molecules may contribute to CSC/LSC resistance, and all three have been considered as potential targets of therapy in solid tumors and leukemias [7,14,16,28,198,201,202].

In the past 15 years, several of the driver kinases have been identified as major targets of therapy. The highlighting example is CML where BCR/ABL-targeting tyrosine kinase inhibitors (TKI) induce major and long-lasting responses [203]. Other similar treatment concepts are emerging in other types of cancers and leukemias as well as in lymphomas. However, it has also been described that in most tumor models, NSC (CSC/LSC) cannot be eradicated completely using these drugs [11,24,28,130,204] as CSC/LSC often grow and survive independent of the primary (major) driver lesion, such as BCR/ABL in CML [204,205].

During the past few years, several major attempts, supported by next-generation sequencing approaches, have been made to reveal additional molecular lesions and the resulting signaling cascades and to define additional target pathways in CSC/LSC [206,207]. Indeed, a number of different signaling pathways - often shared by normal and neoplastic stem cells - have been described to play a role in the evolution and maintenance of CSC/LSC. Several of these pathways have been implicated in stem cell self-renewal. One of these pathways is the Wnt/ß-catenin pathway. This pathway is involved in the maintenance of self-renewal of NSC in leukemias and melanoma as well as in breast, lung, and liver cancers [119,198,208-211]. The Notch signaling pathway has been implicated in self-renewal of CSC in breast cancer, colon cancer, and glioblastoma [201,212-214]. The hedgehog-signaling pathway is also considered to contribute to self-renewal of CSC in various malignancies, such as glioblastoma, breast cancer, colon cancer, pancreatic cancer, and also in leukemias [215-219]. Other signaling pathways may be involved in the regulation of proliferation, survival, and differentiation of CSC. These pathways include, among others, the PI3 kinase-mTOR pathway, the RAS-RAF-MEK-ERK pathway, or the JAK-STAT pathways [196,220-223].

### Role of the microenvironment and cell-cell interactions

**Figure 1:**
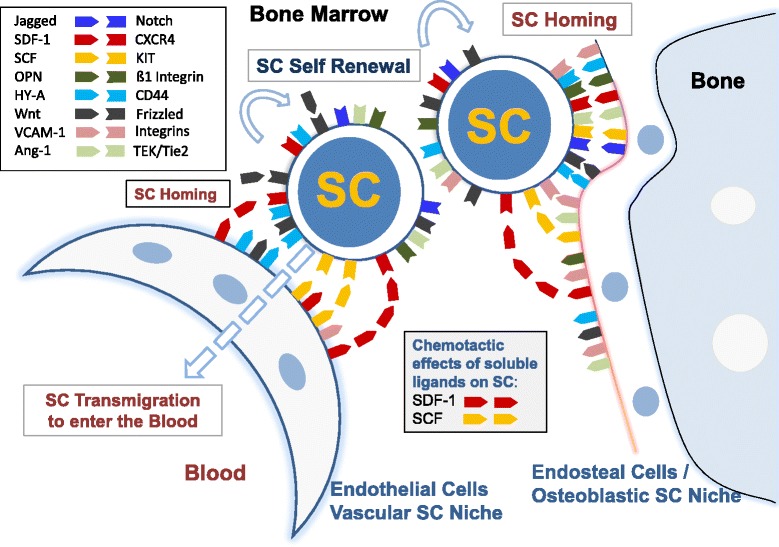
**Cellular interactions in the bone marrow (BM) stem cell niches.** Two types of BM stem cell (SC) niches have been postulated, the vascular SC niche and the endosteal (osteoblastic) SC niche. Both SC niches are considered to play a role in SC homing and SC self-renewal. A number of SC receptors and their ligands regulate qiuesence, self-renewal, proliferation, differentiation, and homing of SC. Relevant ligands are expressed in niche-related cells, including vascular endothelial cells, endosteal cells, and osteoblasts. Whereas several of these ligands are membrane-bound and act as homing receptors, some of them, such as stem cell factor (SCF) or stroma cell-derived factor-1 (SDF-1), can also be produced and released as soluble ligands and thus can act as chemotactic factors for SC. Abbreviations: OPN, osteopontin; HY-A, hyaluronic acid; Ang-1, Angiopoietin-1.

Depending on the stage and type of malignancy, growth and self-renewal of NSC (CSC/LSC) rely on a permissive microenvironment, the CSC niche [4,14,45,72,224,225]. In an early phase of cancer evolution, the CSC niche may regulate growth and self-renewal of premalignant NSC in a similar or in the same way as that of normal stem cells [54,211-216,226,227]. Relevant molecules contributing to stem cell niche interactions in healthy tissues and in ‘premalignant neoplastic states’ include adhesion molecules, chemotactic factors, cytokines, and growth factor receptors [99,225,228-235] (Figure 1). In addition, the local electrolyte milieu, the Ca^2+^ gradient as well as hypoxia may contribute to stem cell niche interactions and stem cell self-renewal in normal and (pre)malignant conditions [225].

#### Stem cell homing and abnormal spread of NSC/CSC

Depending on the organ system, homing of stem cells is a physiologic process [225,236-239]. Likewise, normal hematopoietic stem cells are detectable in the peripheral blood and undergo homing in various organs. In most solid organs, however, stem cells do not undergo redistribution and homing, unless these cells transform to metastasizing CSC. Stem cell homing of normal hematopoietic stem cells and LSC is a multi-step process and the same holds true for the invasion-metastasis cascade of CSC [240,241]. Several different molecules are involved in the homing and invasion process, including selectins and selectin-ligands, integrins and their receptors, and other cell-cell and matrix-binding molecules [240,242]. However, ordered expansion and redistribution from and into the stem cell niches in various organs is usually deregulated in premalignant NSC and malignant CSC/LSC [84,99,225,228-235]. In the normal and leukemic bone marrow, several specific molecular interactions that may contribute to stem cell homing (to the niche) have been identified. These include, among others, SDF-1-CXCR4 interactions, SCF-KIT interactions, and Notch-Notch-ligand interactions (Figure 1) [64,139,225]. In solid tumors, interactions between CSC and the CSC niche are less well defined. One important type of molecules may be cytoadhesion receptors, including integrins, selectins, CD44, or members of the cadherin family. Most of these homing receptors, including chemokine receptors and ligands of matrix molecules such as L1 or CD44, have been detected on CSC [228,229,243]. Likewise, L1 is expressed on the edges of invasive colon cancers and its metastases [230,231] and the same holds true for CD44 and CD133, suggesting that these molecules play a role in tumor invasion and thus disease progression [99,230-232].

During progression of a tumor or leukemia, CSC/LSC may no longer depend on their interaction with the (original) organ-specific microenvironment (CSC niche). Rather, CSC/LSC often expand and redistribute from local sites to other organs to cause metastasis. In epithelial tumors, CSC redistribution is facilitated by the so-called epithelial-mesenchymal transition (EMT), a process that is associated with a loss of specific (adhesive) interactions between cancer cells and the surrounding microenvironment [233,244,245]. Several different molecules, including E-cadherin and L1, have been implicated in the process of EMT in solid tumors [230-233]. Since EMT may also involve CSC, metastasis formation is directly linked to EMT. In hematopoietic neoplasms, similar mechanisms may apply during disease evolution. However, so far, little is known about specific alterations in CSC niche interactions in these malignancies. In CML, LSC have been described to exhibit an adhesion defect that may explain the LSC escape from the bone marrow niche, and subsequent extramedullary spread of progenitors, which is a pathognomonic finding in this type of leukemia [234,235,246].

#### The endosteal and the vascular stem cell niche in the bone marrow

In the normal bone marrow (BM) and in hematopoietic neoplasms, two types of stem cell niches have been postulated, a vascular niche and an endosteal (osteoblastic) stem cell niche (Figure 1). Both niches are considered to act together and thereby trigger self-renewal, proliferation, migration, and redistribution of normal and neoplastic (leukemic) stem cells [225,247-249]. Whereas the endosteal niche is considered to regulate self-renewal and quiescence of normal and neoplastic stem cells, the vascular niche is considered to regulate self-renewal, redistribution, and the leukemic spread of these cells. The postulated vascular niche may primarily be composed of endothelial (arterial) cells and perivascular cells, whereas the endosteal stem cell niche is primarily represented by endosteal-lining cells and osteoblasts [225,247]. The endosteal niche is considered to provide a more hypoxic and hypercalcemic milieu than the vascular niche, which may also contribute to stem cell niche interactions [14,225,250-252] (Figure 1). Several different adhesion molecules, like hyaluronic acid, Jagged, N-cadherin, osteopontin, CAMs, VEGF, SCF, or SDF-1, are considered to contribute to stem cell homing in the niche [225,247-249]. Normal and neoplastic stem cells express receptors for these stromal ligand receptors (Figure 1).

#### Role of hypoxia

Hypoxia and hypoxia-inducible factors (HIF) may influence the fate and self-renewal capacity of stem cells in the micro-milieu of the stem cell niche in health and disease [14,225,250-254]. So far, little is known about the mechanisms through which hypoxia regulates self-renewal and proliferation of CSC. One important aspect may be that hypoxia upregulates not only HIF expression but also several angiogenic and growth-regulatory cytokines, such as SDF-1 (CXCR4) or VEGF [250,255,256]. These cytokines may promote tumor-associated angiogenesis. It has also been described that hypoxia maintains a more stem cell-like state of progenitor cells in the BM by regulating key signaling pathways responsible for stem cell growth and survival, such as Notch or Oct4 [253,254,257,258]. This may also hold true for CSC/LSC in hypoxic areas in the centers of solid tumors [259]. Another important aspect is that hypoxia can trigger the production of reactive oxygen species (ROS) in neoplastic (stem) cells, which in turn leads to DNA breaks and thereby increases mutagenesis and thus the generation of more malignant subclones [260,261]. Thus, hypoxia may be a trigger of oncogenesis and malignant progression as well as CSC/LSC resistance [262-264].

### Plasticity and subclone formation of NSC (CSC/LSC)

**Figure 2:**
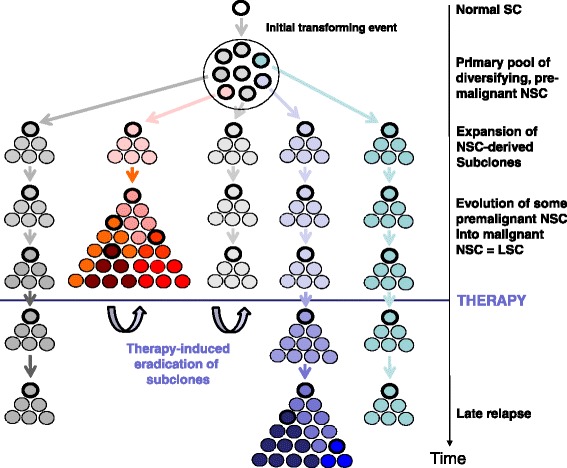
**Subclone formation of CSC during evolution of a malignancy.** During cancer/leukemia evolution, a large number of different subclones with varying combinations of mutational lesions develop. Each change in color is indicative of the acquisition of a relevant new molecular lesion. After a certain time, one or more malignant (dominant) subclones expand and develop into an overt malignancy. However, at the time of diagnosis of a cancer/leukemia, all the other premalignant subclones and their stem cells are also still present. Neoplastic stem cells are indicated by bold circles. After intensive therapy, many or most (sometimes all) of the cancer/leukemic stem cells may have been eradicated. However, the less malignant (pre-malignant) neoplastic stem cells may still survive (because of their quiescence and other resistance-related mechanisms) and may later expand and produce a relapse. Such late relapses may not necessarily express the same oncogenic lesions (driver mutations) compared to the original subclone but still are derived from the same initial stem cell clone. Today, the subclonal architecture is demonstrable by deep sequencing technologies in various malignancies.

A remarkable aspect in the biology of neoplastic stem cells is plasticity and subclone formation during disease evolution which is relevant clinically as subclone formation is often associated with progression and drug resistance. Recent data suggest that in AML and CML, subclone formation is an early and frequent event in LSC development, and the same may hold true for other neoplasms, including solid tumors [26,54,128,129,131,206,220,265]. Plasticity is best explained by genetic instability. The excessive plasticity and subsequent formation of neoplastic subclones is somehow contradictory to the hypothesis that many (at least premalignant) NSC are quiescent cells. However, subclone formation is now considered to be a step-wise and long-lasting process, which may explain the formation of multiple CSC subclones with varying proliferative capacity (Figure 2) [28,48,54,128]. Subclone formation and plasticity of LSC in CML may also be associated with lineage commitment and differentiation or even a lineage switch. One good example is lymphoid or biphenotypic (mixed) blast crisis in Ph + CML [266-269]. In rare cases, subclone formation from LSC is excessive and may result in the development of two histologically unrelated but still monoclonal neoplasms [270-272]. Finally, it has also been reported that some of the hematopoietic neoplasms produce their own (clonal) microenvironment [273-275]. A related observation is ‘vasculogenic mimicry’ that involves the so-called ‘malignant stromal cells’ or ‘malignant endothelial cells.’ Such stromal cell progenitors have recently been detected in several malignancies, including AML [276]. All these observations suggest that the leukemia-associated microenvironment, including the LSC niche, is a new emerging target of therapy.

### Expression of molecular targets in NSC/CSC

An essential question in CSC research is whether certain therapeutic targets are expressed in or on CSC. Notably, targeting of CSC using drugs that can kill or permanently suppress these cells may be a pre-requisite for the development of new curative treatment approaches in cancers and leukemias [7,11,14,16,28]. However, unfortunately, in many instances, CSC and normal stem cells share the same target antigens [64]. As a result, CSC-targeting therapies often result in the occurrence of substantial adverse side effects such as prolonged cytopenia. In this regard, it is noteworthy that the only available curative drug-therapy in AML, which is polychemotherapy, is usually also associated with prolonged cytopenia. Therefore, current research is seeking novel markers and targets that are preferentially or even selectively expressed on CSC (LSC) but are not expressed (or less abundantly expressed) by normal stem cells [67,84,87].

**Figure 3:**
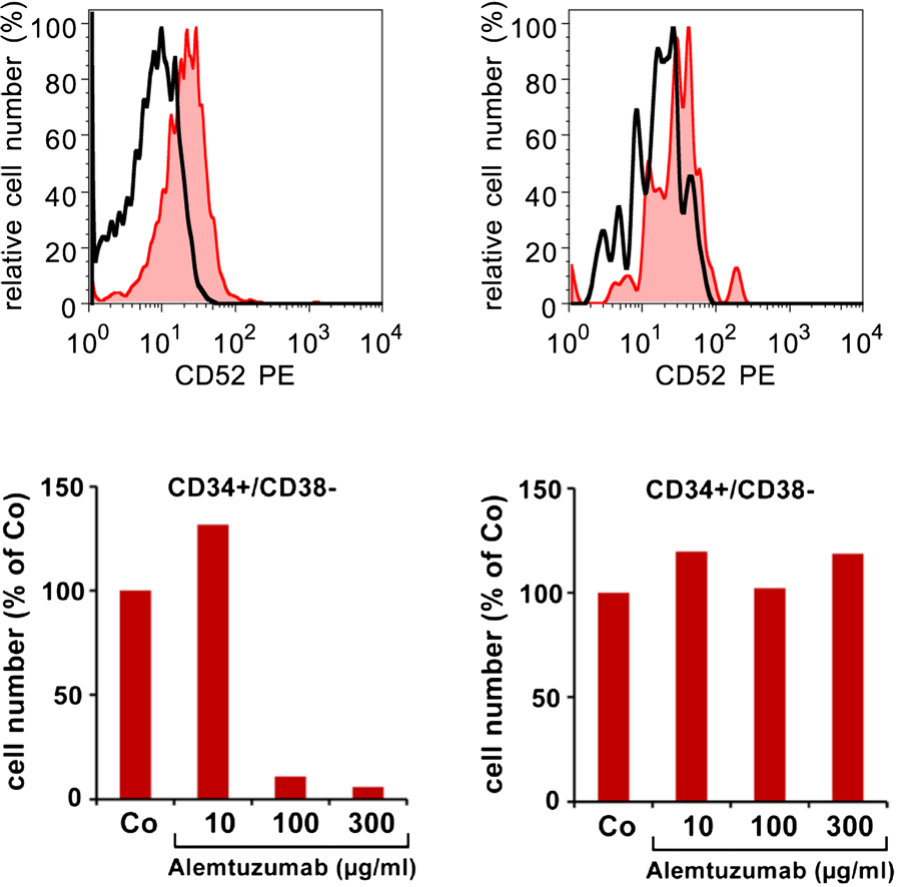
**Leukemic stem cells express the cell surface target antigen CD52.** Upper panels: bone marrow (BM) cells obtained from a patient with acute myeloid leukemia (AML; left panel) or control BM (right panel) cells were stained with antibodies against CD34, CD38, and CD52. The immature CD34+/CD38− stem cells were found to co-express CD52 (red histogram) in the patient with AML but did not express CD52 in the normal BM. The black open histogram represents the isotype-matched control antibody. Lower panels: BM cells were incubated in various concentrations of the CD52-targeted antibody alemtuzumab at 37°C for 1 h. Thereafter, the numbers of viable CD34+/CD38− stem cells were counted by flow cytometry using calibration beads. As visible, exposure to alemtuzumab resulted in a dose-dependent decrease in AML stem cells (left panel) but did not result in a decrease of normal BM stem cells (right panel).

Examples for surface markers/targets that have been described to be expressed primarily on LSC in myeloid leukemias, but less abundantly (or not at all) on normal stem cells, are CD25, CD26, CD33, CD47, CD52, CD96, CD123, IL-1RAP, and CLL-1 [65-69,72,73,85,277-280]. With regard to CD33 and CD52, clinically established targeting concepts are available [280-282]. Likewise, as assessed by *in vitro* and *in vivo* experiments, the CD52-targeting antibody alemtuzumab is able to kill LSC in AML and MDS [77]. Figure 3 shows the effect of alemtuzumab on AML LSC *in vitro*. However, unfortunately, normal stem cells also express low but detectable amounts of these surface antigens, and the respective drugs, gemtuzumab ozogamicin (GO, anti-CD33) and alemtuzumab (anti-CD52) have recently been removed from the oncologic market because of their toxicity profiles which may indeed result in part from their effects on normal stem cells [280-282]. There are also other antibody-based targeted drugs that are currently being developed, such as (among others) CLL-1, IL-1RAP, CD44, CD96, or CD123. The value of these agents is currently being tested preclinically and in clinical trials [283].

A number of different signaling molecules and survival molecules have been identified as potential targets in LSC/CSC. Among these are the PI3K, mTOR, MEK, Smoothened, Notch, Wnt, heat shock proteins, and Bcl-2 family members. Table 5 provides an overview of molecular targets expressed in CSC and LSC in various malignancies. During the past few years, several potent targeted drugs directed against the primary dominant oncoproteins of various tumors and leukemias have been developed. An interesting example is CML, where BCR/ABL blockers are applied successfully to suppress the growth and expansion of LSC [203]. However, even BCR/ABL TKI may not be capable of suppressing all LSC for a prolonged time period, because of stem cell resistance [11,19-23,204]. Nevertheless, the effects of BCR/ABL TKI in CML are a highlighting example of LSC suppression. Notably, in many patients in whom TKI treatment has led to a complete continuous molecular response, treatment discontinuation can be performed, and only a subset of these patients relapse whereas others remain BCR/ABL-negative over years, suggesting that many (clinically relevant) LSC had been eradicated [284].

**Table 5:** **Molecular targets detectable in neoplastic stem cells (NSC)**

**Target type**	**Molecular target example**	**Potentially relevant as NSC-target in**	**Targeted drug example**
Surface antigens	CD20	ALL, CLL	Rituximab [285,286]
	CD33	CML, AML	GO [85,282]
	CD44	AML	mAb [72]
	CD52	5q-AML, CLL	Alemtuzumab [77,287]
	CD123	AML	mAb [69]
	EGFR	Colon-Ca	Cetuximab [288]
	ERBB2	Breast/Gastric/	Trastuzumab [172]
		Ovarian-Ca	
Cytokine receptors	KIT	GIST, CML	Imatinib [203,289]
	PDGFRA	CEL, GIST	Imatinib
	EGFR	Pancreas-Ca	Erlotinib [182]
	ERBB2	Breast-Ca	Lapatinib[290]
Signaling molecules	Hedgehog	Basal cell carcinoma	Vismodegib [291]
	BRAF	Melanoma	Vemurafenib [292]
	BTK	CLL	Ibrutinib [293]
	mTOR	Glioblastoma,	Temsirolimus [294,295]
		Renal cell carcinoma	
Transcription factors	MYC	AML	JQ1 [296]
Niche-NSC-axis	CD26/DPPIV	CML	Gliptins [84]
	CD184/CXCR4-	Plerixafor [159]	
	VEGF-VEGFR	-	Bevacizumab [297]
	SCF-KIT-axis-	Imatinib [203]	

Abbreviations: ALL, acute lymphoblastic leukemia; CLL, chronic lymphocytic leukemia; CML, chronic myeloid leukemia; AML, acute myeloid leukemia; mAb, monoclonal antibodies, EGFR, epidermal growth factor receptor; Ca, carcinoma; GIST, gastrointestinal stroma cell tumor; CEL, chronic eosinophilic leukemia; PDGFR, platelet derived growth factor receptor; BTK, Bruton’s tyrosine kinase; mTOR, mammalian target of rapamycin; MDR-1, multidrug-resistance protein 1; CSA, cyclosporine A; DPPIV, dipeptidyl-peptidase IV; VEGF, vascular endothelial growth factor; SCF, stem cell factor.

### Intrinsic and acquired resistance of NSC/CSC

Normal and neoplastic stem cells benefit from several repair mechanisms and defense systems through which these cells can escape or survive various stress reactions, toxin-exposure, or microbial attacks, and the same mechanisms are responsible for drug resistance [11,19-23,28,298,299]. In the context of neoplastic stem cells, intrinsic forms and acquired forms of resistance have been described. Intrinsic resistance is usually detectable in all CSC populations (subclones), including premalignant NSC and CSC/LSC, whereas acquired resistance is usually found in newly generated, more malignant, subclones and their (subclone-specific) CSC/LSC in advanced neoplasms [11,19-23,298,299].

The mechanisms underlying intrinsic resistance of LSC/CSC are poorly understood. In most neoplasms, multiple factors and mechanisms may act together to produce intrinsic resistance. One factor may be stem cell quiescence [11,19-23,298,299]. Another important factor are cytokine interactions and cell-cell interactions in the CSC niche [14,19-23,28,54]. Moreover, certain drug transporters are expressed differentially in CSC/LSC when compared to more mature neoplastic cells [300-304]. These transporters may mediate drug uptake (such as OCT-1, a drug transporter for Imatinib) but may also contribute to enhanced drug efflux from CSC/LSC. Likewise, in advanced leukemias, LSC often express MDR-1 and probably other drug efflux transporters [22,300-305]. Similar drug transporters have also been identified in solid tumors and in solid tumor CSC. Other mechanisms underlying intrinsic resistance of LSC/CSC may be an abnormal expression or upregulation of survival-related (stress) molecules (often after drug exposure), abnormal expression of signaling molecules or transcription factors, and the lack or loss of tumor suppressor genes or death regulators [11,19-23,28,41,306-312] (Table 6). In addition, the local organ-specific microenvironment, tissue hypoxia, and the interaction with the ‘CSC niche’ may contribute to the resistance of CSC/LSC [11,19-23,28,45,313].

**Table 6:** **Mechanisms of drug resistance in NSC and strategies to overcome resistance**

**Mechanism**	**Proposed strategy to overcame resistance**	**Examples**
NSC quiescence	Antibody-based killing of NSC	CD20, CD33, CD52,
		Bi-specific mAb [77,280,286]
	Mobilization of the immune system against NSC	Vaccination [314],
		IL-2 + histamine [315]
	NSC mobilization into the cell cycle	Cytokine-priming [316]
	CTLA-4 inhibition	Ipilimumab [317]
	PD1 inhibition	Nivolumab [318]
NSC-niche interactions	NSC mobilization out of the niche	Plerixafor [159]
	Redirection of NSC into the niche	Gliptins (CML [84])
	Targeting of niche cells	Revlimid [319]
	Targeting of Niche modulating-cytokines (for example, VEGF) or	Avastin [320]
	Cytokine (for example, VEGF) synthesis	Rapamycin [295]
Enforced drug efflux Verapamil [321,322]	Blocking the efflux pumps	CSA
Expression of anti-apoptotic proteins	Blocking BCL-2 family members	Obatoclax [323]
	Blocking heat shock proteins (Hsp)	Hsp70, Hsp90 [324]

NSC, neoplastic stem cells; IL-2, interleukin-2; CSA, cyclosporine A; CML, chronic myeloid leukemia.

A number of different mechanisms may underlie acquired drug resistance in CSC/LSC. One is genetic instability and the ‘mutation capacity’ of the malignant genome, resulting in a plethora of mutations in critical target genes that can be detected in (more) malignant subclones in these patients [28,54,78,129,206,207,325]. These mutations may occur in an early phase (or even prephase) of the disease. They may develop in most, many, or only a few subclones and may either be detectable at diagnosis (prominent subclone/s) or they remain undetectable for a longer time period because they develop in slowly cycling NSC that are only be capable of generating small-sized subclones [26,28,54,128,325]. Nevertheless, as soon as these small-sized subclones acquire a sufficient number of additional hits (mutations), they can expand and develop into an overt disease in which neoplastic cells and CSC exhibit acquired resistance [26,28,54,128,325]. The use of targeted drugs must lead to a selection of these more malignant subclones over time. Mutations leading to drug resistance may occur in a number of different genes. Likewise, mutations in various tyrosine kinases may contribute to resistance against oncoprotein-targeting drugs [326-328]. The best studied model is CML, where multiple mutations in the BCR/ABL kinase domains have been identified in Imatinib-treated patients [326-328]. Such mutations have been detected in virtually all oncogenic kinases that play a key role in human leukemogenesis or myeloproliferation and also in most other tumor models [329].

Other mechanisms of acquired resistance include the amplifications of target genes (overexpressed targets) or activation of additional pro-oncogenic molecules (Table 5) [330-334]. These types of resistance are usually associated with a poor prognosis and are often accompanied by cytogenetic evidence of clonal evolution. Likewise, in CML and AML as well as in MDS, a complex karyotype usually indicates an unfavorable prognosis [334-336].

### Can we translate the CSC concept into clinical practice?

Most of the conventional anti-cancer agents currently used in daily practice or in clinical trials are primarily acting on rapidly dividing cells that make up the bulk of the tumor, whereas most CSC (and premalignant NSC) are not affected. High-dose chemotherapy and novel targeted drugs may be able to eliminate the bulk of the neoplasm and to eradicate most CSC (or LSC) in a given tumor or leukemia. These debulking agents are still very useful and instrumental in anti-cancer therapy. However, relapses may develop from a few residual, drug-resistant, premalignant (quiescent) NSC that exhibit intrinsic stem cell resistance. Notably, even if all CSC/LSC can be eradicated by drug therapy, (late) relapses can develop from such residual, mostly quiescent premalignant NSC [26,28,54,128,131,220,325]. In other words, many new drug therapies can eliminate the mass of CSC/LSC that have generated the dominant clone but are unable to eradicate all quiescent premalignant NSC forming smaller subclones [26,54,128,284]. These drugs may even lead to operational cures without having the potential to eradicate the disease completely [128,284]. The question is how relevant the residual (often quiescent) NSC are in these patients. Notably, not all types of MRD and MRD-specific NSC may be relevant clinically, even if they may expand to another dominant clone [28,54,128]. Likewise, in hairy cell leukemia, cladribine (2CdA) may not be able to eradicate all LSC, and most premalignant NSC may survive. However, because of the relatively slow growth rate and low mutation rate of NSC, full blown relapses are relatively uncommon; and if they occur (typically after 3 to 5 years), leukemic cells are again responsive to the same drug. By contrast, in AML, the mutation rate is high and relapses are always indicative of a poor outcome and are often associated with multidrug resistance. The same holds true for most solid tumors. In CML, several novel TKI may induce complete continuous molecular remissions (CMR) [337,338]. Even imatinib can induce long-term CMR in a smaller fraction of patients [284]. When TKI are discontinued in these patients, some of them will relapse but may again respond to imatinib or other new TKI [284]. The exact curative potential of imatinib and of the new TKI in CML remains unknown. In solid tumor, novel TKI have also been applied in clinical trials and some of these agents are rather promising. However, long-term remissions are usually not induced with these agents even when combined with chemotherapy. Overall, with a few exceptions, in most advanced solid tumors, no drug-based CSC-eliminating treatment approach has been developed so far. However, there are several examples where targeted drugs as single agents may lead to long-term disease control. One example is the gastrointestinal stroma cell tumors (GIST), where TKI have shown encouraging results [339-341]. Another example is renal cell carcinoma, where inhibitors of the PI3K-mTOR pathway have shown to exert major anti-tumor effects [342,343].

A general problem in cancer evolution is that many CSC/LSC and most or all premalignant NSC may be dormant cells, and that dormancy is often associated with intrinsic resistance. One possible way to overcome this type of resistance may be to apply targeted antibodies, especially antibody-toxin conjugates which often act independent of the cell cycle and thus can destroy even dormant NSC. Likewise, in several types of lymphomas, the addition of pan-B-cell-targeting antibodies has substantially improved cure rates and the overall outcome (survival) in these patients [287,344,345]. An alternative strategy is to mobilize dormant cells into the cell cycle or out of the niche (where dormancy may be propagated) [159,346]. Finally, dormancy of NSC/CSC may be overcome by exposure to cytokines that promote cell cycle progression in NSC/CSC. Another principal strategy may be to promote CSC/LSC exhaustion by inducing differentiation and maturation in these cells or by mobilizing the immune system against CSC/LSC.

A major problem is that in advanced cancer lesions, CSC not only exhibit intrinsic (natural) stem cell resistance but often also acquired drug resistance in more resistant and thus more malignant subclones [28,54,128]. One strategy to address the multiple mechanisms of resistance accumulating in advanced tumor lesion is to apply drug combinations. Another strategy is to combine conventional or targeted drugs with response modifiers or agents that mobilize tumor cells into the cell cycle. An alternative approach is to select targeted drugs that can overcome acquired drug resistance resulting from point mutations in critical target genes. Likewise, in CML, second-generation BCR/ABL TKI can often overcome imatinib resistance associated with BCR/ABL mutations [338,347,348].

Another aspect in CSC/LSC evolution is that resistance of CSC/LSC is often associated with specific interactions between these cells and CSC niche. One strategy to overcome this form of resistance is to mobilize CSC/LSC from the niche where stem cells are considered to be protected and thus less accessible to targeted drugs. One example is the SDF-1/CXCR4 axis that can be disrupted by the CXCR4 blocking agent Plerixafor [349,350]. Recent data suggest that Plerixafor cannot only mobilize normal hematopoietic stem cells from the bone marrow stem cell niche but also LSC and that Plerixafor-mobilized LSC may be more sensitive against certain anti-leukemic drugs [159,346]. However, it remains unknown whether all LSC can be mobilized by Plerixafor, whether the mobilization is associated with a rebound of more rapidly growing LSC in the niche and whether addition of Plerixafor to conventional chemotherapy will indeed increase response and cure rates in patients with AML or other leukemias. In addition, more recent data suggest that in certain forms of leukemias (CML), LSC are already mobilized cells that can easily traffic between niches [84].

Recent data suggest that the vascular (arteriolar) stem cell niche is of particular importance for self-renewal of LSC [225,247-249]. Therefore, additional effects of targeted drugs on vascular cells or drug combinations employing anti-angiogenic agents are of considerable interest. One of these anti-angiogenic drugs is Lenalidomide, a major anti-angiogenic drug that produces major responses in various myeloid and lymphoid neoplasms, including 5q-MDS, multiple myeloma, and chronic lymphocytic leukemia. Several other anti-angiogenic drugs have been tested in hematopoietic and solid malignancies, with varying success. Encouraging results have been obtained when combining these agents with other anti-neoplastic agents. A remarkable observation is that the novel BCR/ABL TKI Nilotinib and Ponatinib (but not imatinib) are potent inhibitors of endothelial growth and angiogenesis [351]. Whether these additional effects of these TKI are responsible for their better efficacy in CML remains unknown. This is an attractive hypothesis, since their much stronger effects on BCR/ABL (when compared to imatinib) fail to explain their excellent clinical efficacy, as CML LSC are considered to survive independent of BCR/ABL [352,353]. Table 6 provides strategies aimed at overcoming LSC resistance in human malignancies.

### Summary and future perspectives

During the past few years several CSC/LSC-targeting concepts have been developed with the aim to establish more effective treatment approaches in applied oncology. However, although such novel treatment concepts are straight-forward, several questions remain. First, the complexity of the somatic aberration networks and of the resulting signaling cascades that drive oncogenesis during CSC/LSC evolution and may lead to CSC/LSC resistance. To address this aspect, the use of drug combinations or broadly acting drugs has been suggested and may be required to eliminate or suppress all relevant CSC/LSC populations in a given neoplasm. Novel treatment concepts have to take additional aspects into account, including intrinsic resistance, the related issue of CSC/LSC quiescence, and the interaction of CSC/LSC with their organ- and disease-specific microenvironment (CSC niche). Additional local factors such as hypoxia may also play a role in CSC/LSC resistance. The hope for the future is that we will be able to exploit our increasing knowledge about CSC/LSC, in order to define new treatment concepts, with the ultimate aim to eradicate CSC/LSC in various cancer types as well as in leukemias. In advanced, drug-resistant neoplasms, such treatment concepts may need to be combined with high-dose chemotherapy and/or stem cell transplantation.

## Abbreviations

BM: bone marrow
CD: cluster of differentiation
CML: chronic myeloid leukemia
CSC: cancer stem cell(s)
CXCR-4: C-X-C chemokine receptor type 4
EMT: epithelial-mesenchymal transition
FGF: fibroblast growth factor
G-CSF: granulocyte colony-stimulating factor
GM-CSF: granulocyte-macrophage colony-stimulating factor
Her 2: human epidermal growth factor receptor 2
IL: interleukin
LSC: leukemic stem cell(s)
NOD/SCID: non-obese diabetic mice with severe combined immunodeficiency
NOG: IL-2Rgamma chain-knock out NOD/shi-SCID mice
NSC: neoplastic stem cell(s)
NSG: NOD/SCID with loss-of-function-mutated IL-2Rgamma chain
PI3K: phosphatidylinositide-3 kinase
SCF: stem cell factor
SDF-1: stromal cell-derived factor 1
TKI: tyrosine kinase inhibitor(s)
